# Effects of using coding potential, sequence conservation and mRNA structure conservation for predicting pyrrolysine containing genes

**DOI:** 10.1186/1471-2105-14-118

**Published:** 2013-04-04

**Authors:** Christian Theil Have, Sine Zambach, Henning Christiansen

**Affiliations:** 1 Research group PLIS: Programming, Logic and Intelligent Systems, Department of Communication, Business and Information Technologies, Roskilde University, P.O. Box 260, Roskilde, DK-4000, Denmark

## Abstract

**Background:**

Pyrrolysine (the 22nd amino acid) is in certain organisms and under certain circumstances encoded by the amber stop codon, UAG. The circumstances driving pyrrolysine translation are not well understood. The involvement of a predicted mRNA structure in the region downstream UAG has been suggested, but the structure does not seem to be present in all pyrrolysine incorporating genes.

**Results:**

We propose a strategy to predict pyrrolysine encoding genes in genomes of archaea and bacteria. We cluster open reading frames interrupted by the amber codon based on sequence similarity. We rank these clusters according to several features that may influence pyrrolysine translation. The ranking effects of different features are assessed and we propose a weighted combination of these features which best explains the currently known pyrrolysine incorporating genes. We devote special attention to the effect of structural conservation and provide further substantiation to support that structural conservation may be influential – but is not a necessary factor. Finally, from the weighted ranking, we identify a number of potentially pyrrolysine incorporating genes.

**Conclusions:**

We propose a method for prediction of pyrrolysine incorporating genes in genomes of bacteria and archaea leading to insights about the factors driving pyrrolysine translation and identification of new gene candidates. The method predicts known conserved genes with high recall and predicts several other promising candidates for experimental verification. The method is implemented as a computational pipeline which is available on request.

## Background

Over the past two decades, the standard genetic code has been revised to include the two new amino acids, selenocysteine and pyrrolysine. These amino acids are, under certain circumstances, encoded by codons that are normally stop codons. Translation of these codons can be influenced by the mRNA structure. This is the case for selenocysteine where a cis-acting mRNA structure (SECIS) drives translation of the opal stop codon (UGA) as selenocysteine. Similarly, a structure (PYLIS) has been identified in some genes where pyrrolysine is encoded by the (usual) stop codon UAG [1]. The structure lies in the region between the UAG codon and approximately 100 bp downstream. The role of the structure in translation is unclear and it is only conserved among some pyrrolysine incorporating genes [2]. Zhang et al. [2] suggest that either a complete recoding of the UAG codon as pyrrolysine occurs or alternatively that UAG serves a dual function in pyrrolysine incorporating organisms; termination and translation competes leading to "statistical proteins" where both terminated and elongated products occur, but where the amounts of protein products may depend on circumstances.

The latter possibility is substantiated by an in vitro study [3] where the components necessary for pyrrolysine synthesis are inserted into E. coli. The study shows that the PYLIS structure is not essential for translation of pyrrolysine incorporating genes, but also concludes that the presence of the structure results in a higher amount of pyrrolysine incorporating protein products and that synonymous codon mutations in the PYLIS sequence result in lesser amounts.

The translation of pyrrolysine is associated with methane metabolism. All known organisms with methane metabolism have pyrrolysine incorporating methyltransferases, that initiate the transfer of methyl groups from methyl amines and into a process of which methane is the result [4,5]. Three distinct methyl trans-ferases have been identified — monomethylamine methyltransferase (mtmB), dimethylamine methyltrans-ferase (mtbB), and trimethylamine methyltransferase (mttB) — each of which allows metabolism of different kinds of methyl amines [6]. Each transferase catalyzes the transfer of a methyl group from mono-, di- or trimethylamine to each of their respective corrinoid cofactors, and the three methyltransferases are not all present in all methane producing organisms. It has been hypothesized that the availability of methyl amines regulates translation of UAG as pyrrolysine [2].

While selenocysteine is translated in a broad variety of organisms including archaea, bacteria and eukaryotes, pyrrolysine translation was up until recently believed to occur only in a few microbes, but it has recently been observed in a somewhat larger number of genomes within archea and bacteria [7]. So far, approximately 16 species are known to have pyrrolysine-containing genes.

Methods for identification of selenocysteine encoding genes based on detection of the SECIS structural motif are quite successful [8]. Such methods may successfully identify genes with a PYLIS structure, but have difficulties predicting pyrrolysine incorporating genes without the consensus structure. Previous com-putational methods for detection of pyrrolysine genes, e.g., [9,10], are based on homology search. These methods do not consider structural conservation and codon sequence composition of downstream regions for predicting pyrrolysine incorporating genes. In this paper we introduce an approach which takes all these factors in account. Our approach does not assume a particular consensus structure to be present, but is capable of taking conserved structure in the region downstream UAG into account.

## Methods

**Figure 1:**
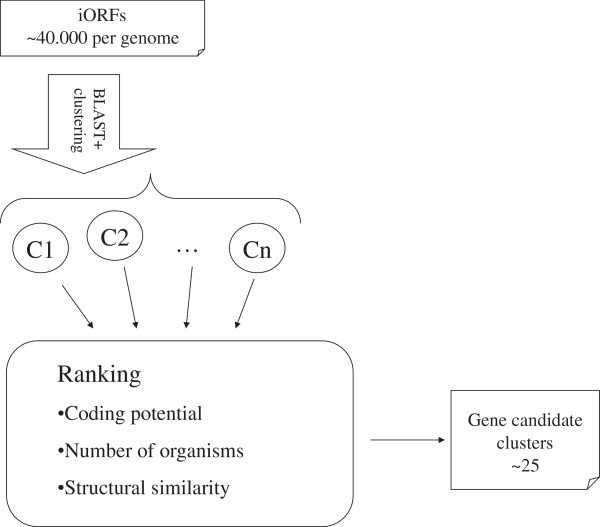
**Illustration of the pipeline for identifying pyrrolysine containing genes.** The process extracts iORFs which are then clustered using blast. Finally, the clusters are ranked according to several features.

An flowchart illustrating our method is shown in Figure 1. Details of the steps involved are described in the following subsections.

### Identification of relevant organisms

We identify organisms of interest by searching for a tRNA^pyl^ synthetase using blast [11]. Additionally, we search for the tRNA^pyl^ by creating a structure profile of the tRNA^pyl^ using ClustalW [12] and RNAalifold [13]. This profile is used to screen the genomes with Infernal [14]. The genomes verified to have the tRNA^pyl^ and for which complete assembled genomes are available are used for further investigations. These are, in alphabetical order:

• Acetohalobium arabaticum [RefSeq:NC_014378.1]

• Desulfitobacterium hafniense [RefSeq:NC_011830.1]

• Desulfobacterium autotrophicum [RefSeq:NC_012108.1]

• Desulfosporosinus orientis [RefSeq:NC_016584.1]

• Desulfotomaculum acetoxidans [RefSeq:NC_013216.1]

• Methanococcoides burtonii [RefSeq:NC_007955.1]

• Methanohalobium evestigatum [RefSeq:NC_014253.1]

• Methanohalophilus mahii [RefSeq:NC_014002.1]

• Methanosalsum zhilinae [RefSeq:NC_015676.1]

• Methanosarcina acetivorans [RefSeq:NC_003552.1]

• Methanosarcina barkeri [RefSeq:NC_007355.1]

• Methanosarcina mazei [RefSeq:NC_003901.1]

• Thermincola potens [RefSeq:NC_014152.1]

A summary of the genome screening can be found in Table 1.

**Table 1:** Table of potential Pyl-coding organisms and their pyrrolysine connection

**Organism**	**Family**	**Known Pyl**	**tRNA**^**Pyl**^	**pylS**	**Whole genome avail**	**MtxB gene found**
Methanosarcina acetivorans	Archaea (Methanosarcinaceae)	Yes	Yes[15]	Yes	Yes	Yes
Methanococcoides burtonii	Archaea (Methanosarcinaceae)	Yes	Yes[15]	Yes	Yes	Yes
Methanosarcina barkeri	Archaea (Methanosarcinaceae)	Yes	Yes[15]	Yes	Yes	Yes
Methanosarcina mazei	Archaea (Methanosarcinaceae)	Yes	Yes[15]	Yes	Yes	Yes
Methanohalophilus mahii	Archaea (Methanosarcinaceae)	Yes	Verified by Infernal	Yes	Yes	Yes
Methanohalobium evestigatum	Archaea (Methanosarcinaceae)	Yes	Verified by Infernal	Yes	Yes	Yes
Methanosarcina thermophila	Archaea (Methanosarcinaceae)	Yes	Verified by Infernal	Yes	No	Yes
Methanosalsum zhilinae	Archaea (Methanosarcinaceae)	Yes	Verified by Infernal	Yes	Yes	Yes
Desulfitobacterium hafniense	Bacteria (Clostridia)	Yes	Yes[15]	Yes	Yes	Yes
Desulfitobacterium autotrophicum	Bacteria (Deltaproteobacteria)	Yes	Yes[15]	Yes	Yes	Yes
Desulfotomaculum acetoxidans	Bacteria( Clostridia)	Yes	Verified by Infernal	Yes	Yes	Yes
Bilophila wadsworthia	Bacteria (Deltaproteobacteria)	Yes	-	Yes	No	Yes
Acetohalobium arabaticum	Bacteria (Clostridia)	Yes	Verified by Infernal	Yes	Yes	Yes
Thermincola potens	Bacteria (Clostridia)	Yes	Verified by Infernal	Yes	Yes	Yes
Desulfosporosinus orientis	Bacteria (Clostridia)	-	Verified by Infernal	Yes	Yes	Yes
Desulfotomaculum gibsoniae	Bacteria (Clostridia)	-	-	Yes	No	Yes
Desulfosporosinus meridiei	Bacteria (Clostridia)	-	-	Yes	No	Yes
Geodermatophilus obscurus	Bacteria (Actinobacteria)	-	Verified by Infernal*	-	No	-

The organisms marked in bold are used in further analyses. We exclude the organisms for which a whole genome sequence is not currently available. We furthermore exclude organisms which do not have both a transfer RNA with UAG anticodon (tRNA^pyl^) and a corresponding tRNA synthetase (pylS).

* No UAG-anticodon.

### Extraction of interrupted ORFs

We adopt the terminology Interupted ORFs (iORFs) from Chaudhuri and Yeates [9] and in a similar vein, we extract iORFs from the genomes of interest. Interrupted ORFs are like traditional ORFs except that they contain an UAG codon between the first potential start codon and the following stop codon. Such iORFs are described by the following grammar in Extended Backus-Naur Form [16],

<iORF> ::= < start > <not-stop > * < amber > <not-stop > * < stop>

<start> ::= TTG | CTG | ATT | ATC | ATA | ATG | GTG

<stop> ::= TAA | TAG | TGA

<amber> ::= TAG

<regular> ::= AAA | … | TTT //*all codons except those in* < start > *and* < stop>

<not-stop> ::= < start > | < regular>

An iORF is any subsequence of nucleotides specified by the grammar in either the sense strand or the reverse complemented anti-sense strand. The star notation in the first rule indicates a repetition (zero or more times), and the vertical bar is used for alternatives.

We extract only iORFs that have at least 100 bases downstream the UAG. This ensures that the iORF can accommodate a PYLIS structure. An obvious consequence of this restriction is that we do not consider iORFs where the PYLIS structure could possibly extend beyond the stop codon or where a hypothetical PYLIS structure occurs upstream the amber codon.

### Reciprocal blast

Presumably, the PYLIS structure region is subject to purifying selective pressure due to both its protein function and the possible importance of a putative structure.

We identify homologous putative PYLIS sequences (100 bp downstream the UAG) conserved at amino acid level using a reciprocal blast search. For each iORF, we translate the region 100 bp downstream the UAG to its amino acid sequence. Then, using tblastn with an e-value threshold of 10^-6^ we search for this amino acid sequence in all the candidate genomes. We disregard hits to the query iORF itself and also hits to non-iORF regions.

The result of this search is a set of pairwise matches between some of the iORFs. Transitively, matched iORFs form connected clusters of similar hits. If there is a blast match between two iORFs, then they are members of the same cluster. An iORF can only be member of one cluster. In graph theoretical terms, an undirected graph is formed such that iORFs constitute nodes and matches between iORFs constitute edges. The clusters are then those connected components of the graph that include more than one node.

We disregard clusters that include an iORF where a) the region 100 bp downstream is completely overlapped by a known gene^a^ in a different reading frame or b) the region partially overlaps a known functional RNA gene.

In particular, requirement a) excludes a lot of shadow ORFs that may arise when a true protein sequence in a different reading frame is conserved. Since we only consider overlaps with genes in different reading frames, iORFs for known pyrrolysine incorporating genes are not eliminated since those will be in the same reading frame as the iORF. It happens that such genes are erroneously annotated as two genes in RefSeq^b^, where the first one uses the UAG as stop codon, but these are still in the same reading frame as the iORF.

After these pruning steps, 1789 clusters remain.

### Feature extraction

Coding potential is a measure of the likelihood that a stretch of DNA may encode a protein. Protein coding genes exhibit a non-random sequence of codons that turn out to be a strong indicator of coding potential. Many contemporary gene finders use variants of Hidden Markov Models (HMM) to statistically model the codon sequences of genes. We apply an HMM in which the hidden states correspond to amino acids. These hidden states can emit the codons that encode the amino acid with distinct probabilities [17]. Additionally the HMM incorporates length modeling of genes. As an adaptation to be able to model iORFs as well as usual ORFs, the states may emit the UAG codon (with no effect on probability) in addition to the usual probabilistic codon emission. This adaptation means that we are able to train the model on non-pyrrolysine incorporating genes, but are able to decode also on iORFs^c^.

We train the model on the RefSeq annotated genes of each genome resulting in maximum-likelihood parameters Θ _*g*_ for each genome *g*.

The trained models are used assign a probability to each iORF *i* from a genome *g*. In effect, the probability reflects how much the iORF *i* resembles the known genes of the genome *g* in sequence composition and length. A log-odds ratio is calculated using the probability score of a nucleotide sequence model *model*_*null*_ as null model, which assumes that all nucleotides occur with the same frequency:

$$\mathrm{HM}M_{g}\left(i\right)=\log\;P\;\left(i\left|\mathrm{mode}l_{\mathrm{HMM}}\Theta_{g}\right)\right)-\log\;P\left(\mathrm{iORF}\;\left|\mathrm{mode}l_{\mathrm{null}}\right)\right).$$

We define the coding potential of a cluster ω of size *n* to be the average of iORF coding potential scores within a cluster,

$$f_{\omega }^{\mathrm{coding}}=\frac{1}{n}*\sum_{i\in \omega }\mathrm{HM}M_{g}\left(i\right),\;i\;\mathrm{is}\;\mathrm{an}\;\mathrm{iORF}\;\mathrm{from}\;\mathrm{genome}\;g$$

Only 958 of the initial 1789 have $f_{\omega }^{\mathrm{coding}}<0$. We only consider these 958 clusters for further investigation. The number of homologues may be indicative of functional importance. We define a feature $f_{\omega }^{\mathrm{size}}$ that measures the number of hits in a cluster, $f_{\omega }^{\mathrm{size}}=\left\|\omega \right\|$. The $f_{\omega }^{\mathrm{size}}$ feature does not distinguish between homologues within or across species. Since conservation among species is a stronger indicator of important function, we also define a feature $f_{\omega }^{\mathrm{Organisms}}$ which is the number of unique organisms present in a cluster *ω*.

We expect clusters that contain real PYLIS regions to be relatively more diverse in their nucleic sequence than in their amino acid sequence, whereas this may not be the case for spurious hits. On the other hand, primary sequence variation can have a degrading effect on protein function and for homologue genes within the same organism, the variation may be minimal. We model diversity using the *f*^*diversity*^ feature, which is calculated as the average distance between the PYLIS regions of all *n* iORFs in a cluster,

$$f_{\omega }^{\mathrm{diversity}}=\frac{1}{n^{2}-n}\sum_{s,t\in \omega ,s\neq t}{\mathrm{DIST}}_{3}\left(s_{\mathrm{pyl}}t_{\mathrm{pyl}}\right)$$

where *s*_*pyl*_ and *t*_*pyl*_ are the regions 100 bp downstream of the in-frame UAG of *s* and *t*, respectively. *dist*_*m*_(*s*_*pyl*_, *t*_*pyl*_) is the edit distance — the smallest number of insertions, deletions or mutations needed to transform *s*_*pyl*_ into *t*_*pyl*_ — disallowing gaps that are not in multiples of *m*. Note that DIST_*m*_ is symmetric, i.e., DIST_*m*_(a, b) = DIST_*m*_(b, a), but for convenience of notation, the feature includes all pairwise distances.

Since primary sequence variation may have a degrading effect on a protein, we also consider the average number of synonymous codons, *f*^*syn_codons*^, defined as,

$$f_{\omega }^{\mathrm{syn}\_\mathrm{codons}}=\frac{1}{n^{2}-n}\sum_{s,t\in \omega ,s\neq t}\frac{1}{{\mathrm{DIST}}_{1}\left({s^{\prime }}_{\mathrm{pyl}},{t^{\prime }}_{\mathrm{pyl}}\right)}$$

where, *s'*_*pyl*_ and *t'*_*pyl*_ are the amino acid sequences translated from *s*_*pyl*_ and *t*_*pyl*_.

Note that *f*^*diversity*^ and *f*^*syn_codons*^ are inversely correlated except in cases where diversity is preferen-tially in third codon position such that it leads to synonymous codons.

The iORF extraction step ensures that iORFs have at least 100 bases downstream an in-frame UAG. In many clusters, however, iORFs have only a few bases upstream the UAG and a start codon just upstream the UAG. In such short upstream regions it becomes more likely that the UAG and upstream start codon occur due to chance. To address this we define the features *f*^*upstream*^ and *f*^*downstream*^,

$$f_{\omega }^{\mathrm{upstream}}=\frac{1}{n}\sum_{i\in \omega }\left\|i_{\mathrm{start}}\ldots i_{\mathrm{uag}}\right\|$$

where ‖*i*_*start*_ … *i*_*uag*_‖ is the distance in nucleotides from the start codon to the in-frame UAG codon and

$$f_{\omega }^{\mathrm{downstream}}=\frac{1}{n}\sum_{i\in \omega }\left\|i_{\mathrm{uag}}\ldots i_{\mathrm{stop}}\right\|$$

where ‖*i*_*uag*_ … *i*_*stop*_‖ is the distance in nucleotides from the UAG codon to the stop codon.

Assuming that the structure of the PYLIS region may be important, we model structural similarity within a cluster *ω* with the feature $f_{\omega }^{\mathrm{structure}}$ defined as follows. We measure similarity based on alignment of base-pairing probabilities of the sequences, which is independent of any predicted structure. We compute the base-pairing probabilities using RNAfold [18] and align these using pmcomp [19] with default settings. The *f*^*structure*^ score is the average pmcomp score for each pair of pylis regions in a cluster,

$$f_{\omega }^{\mathrm{structure}}=\frac{1}{n^{2}-n}\sum_{s,t\in \omega ,s\neq t}\mathrm{PMCOMP}\left(s_{\mathrm{pyl}},t_{\mathrm{pyl}}\right)$$

### Normalization

We normalize features to the interval [0, 1]; ${\hat{f}}_{i}^{j}$ is the normalized value for the *j*’th feature in the *i*’th cluster, defined as

$${\hat{f}}_{i}^{j}=\frac{f_{i}^{j}-\min\left(f^{j}\right)}{\max\left(f^{j}\right)-\min\left(f^{j}\right)}$$

where $f_{i}^{j}$ is the value for the *j*'th feature for the *i*'th cluster, min(*f*^*j*^) is the minimum value for feature in any cluster and vice versa for max(*f*^*j*^).

### Complex features

**Figure 2:**
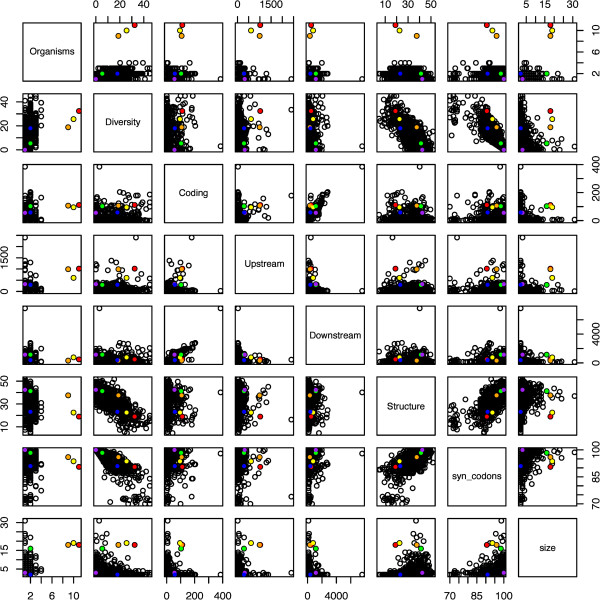
**Correlations between cluster features.** Each cluster is represented as a point in each of the panels. Each panel shows the correlation between a pair of feature values. Known pyrrolysine gene clusters are marked with colors: *mttB* (red), *mtbB* (orange), *mtmB* (yellow), *transposase*_1_ (green), *transposase*_2_ (blue) and *TetR* (purple). Unknown clusters are white. Feature combinations with discriminatory potential display significant separation between the bulk of white clusters (the majority of which are false positives) and the colored clusters (true positives). A separation is apparent in panel 4.3 which shows the combination of the *f*^*coding*^ and *f*^*upstream*^ features.

In addition to the basic features, we derive two combined features based on our intuition and on observed correlations (see Figure 2). *f*^*coding*^ and *f*^*upstream*^ are inversely correlated in general, but positively correlated for the known clusters. The negative correlation occurs, e.g., in the case of an iORF with a long gene just downstream the UAG (high *f*^*coding*^) but a only a few bases upstream the UAG. The correlation effect observed between *f*^*coding*^ and *f*^*downstream*^ is less pronounced. This motivates the addition of a combined feature, $\sqrt{{\hat{f}}^{\mathrm{coding}}\times {\hat{f}}^{\mathrm{upstream}}}$, which is the geometric mean of ${\hat{f}}^{\mathrm{coding}}$ and ${\hat{f}}^{\mathrm{upstream}}$.

Structural similarity may arise by chance. In the absence of sequence diversity, it is diffcult to judge the significance of a structurally similar cluster. To penalize diversity due to overhangs in blast hits and diversity that has a degrading effect on the amino acid sequence, we also take into account the number of synonymous codons. This leads to a combined feature $\sqrt[{3}]{{\hat{f}}^{\mathrm{structure}}\times {\hat{f}}^{\mathrm{diversity}}\times {\hat{f}}^{\mathrm{syn}\_\mathrm{codons}}}$.

### Feature significance

For each of the features we calculate a p-value to assess its statistical significance. We obtain *p*-values in the following way: We sample without replacement 10^5^ means of *n* random ranks out of the 958 possible, where n is the number of clusters containing known pyrrolysine-incorporating genes^d^. Building on the central limit theorem which guarantees that the distribution of means is normal, we fit the sampled means to a normal distribution (*μ* ≈ 479, *σ ≈* 112). *p*-values – probabilities of getting a mean rank at least as low as a given mean rank by chance – can be calculated from the estimated normal distribution by integrating the area below the given mean rank (cumulative density function)^e^.

We calculate the p-value for the mean rank — when ranked according to each feature — of the clusters known to include pyrrolysine incorporating genes.

Features for which the null hypothesis cannot be rejected (*p* > 0:05) are not used for the final ranking, i.e., we use a subset of features indexed by *h*: *p*(*rank*(*f*^*h*^)) > 0:05.

The *p*-values of each feature are reported in Table 2.

**Table 2:** Cluster ranking

	**mttB**	**mtbB**	**mtmB**	**transposase**_**1**_	**tranposase**_**2**_	**TetR**	**p-value**
*f*^*organisms*^	11	9	10	2	1	2	-
*f*^*diversity*^	32.2	18.8	25.2	5.4	0.0	18.0	-
*f*^*structure*^	19.0	37.6	22.5	41.3	42.4	23.2	-
*f*^*coding*^	111.8	107.1	95.2	103.2	56.0	58.3	-
*f*^*upstream*^	1016.6	992.1	605.4	293.2	325.7	297.5	-
*f*^*downstream*^	495	325.4	773.2	1150	1149.7	391	-
*f*^*syn_codons*^	90.6	96	93.7	98.4	100	91.0	-
*f*^*size*^	18	18	19	16	3	2	-
*rank*(*f*^*organisms*^)	1	3	2	84	508	85	**0.0006**
*rank*(*f*^*diversity*^)	32	222	87	639	890	243	0.13
*rank*(*f*^*structure*^)	895	344	858	196	147	840	0.72
*rank*(*f*^*coding*^)	22	26	38	31	80	76	**0.00006**
*rank*(*f*^*upstream*^)	6	7	13	46	32	41	**0.000027**
*rank*(*f*^*downstream*^)	6	90	18	602	888	255	0.064
*rank*(*f*^*syn_codons*^)	793	385	596	252	99	762	0.51
*rank*(*f*^*size*^)	8	9	6	10	305	498	**0.0013**
$\mathrm{ranks}\left(\sqrt{{\hat{f}}^{\mathrm{coding}}\times {\hat{f}}^{\mathrm{upstream}}}\right)$	4	5	10	16	18	19	**0.000017**
$\mathrm{rank}\left(\sqrt[{3}]{{\hat{f}}^{\mathrm{structure}}\times {\hat{f}}^{\mathrm{diversity}}\times {\hat{f}}^{\mathrm{syn}\_\mathrm{codons}}}\right)$	211	6	132	504	876	417	0.13
rank(regression)	2	3	4	15	23	19	**0.000015**

Raw feature values and ranking of known true positive clusters based on single features and combined ranking using regression weights. *p*-values for significant features (p > 0:05) are marked in bold.

### Comparison of prediction results

Based on significant normalized feature values ${\hat{f}}_{i}^{h}$ we calculate a combined cluster score which is used for ranking,

$$\mathrm{scor}e_{i}=\sum_{h}w^{h}{\hat{f}}_{i}^{h}$$

where *w*^*h*^ is a unique weight associated with feature ${\hat{f}}_{i}^{h}$. We estimate weights for each feature using gradient descent to minimize the sum of ranks of positive examples,

$$\underset{w^{1}\ldots w^{n}}{\arg\;\min}\sum_{e\in E+}\mathrm{ran}k^{e}$$

The set of positive examples, denoted E^+^, are clusters that include known pyrrolysine incorporating genes. There are six of these: mtbB, mttB, mtmB, two clusters with transposases only annotated in M. acetivorans, and a transcriptional regulator of the TetR family also only annotated in M. acetivorans. In the genomes we consider there are five other (RefSeq) annotated genes with in-frame UAGs, but these are not conserved and as result, they are not present in reciprocal blast clusters.

The ranking scheme is based on a simple a linear combination of features, where the weights are estimated by regression over the rankings of the known positive examples. It is possible to devise a more precise but complex ranking function, but we have opted for this simple scheme. We have done so because we only have a few positive examples, and there is a large potential for overfitting a more complex function. With the few positive examples available, we have no real means of doing cross-validation and even this simple function may slightly overfit. With the discovery of additional pyrrolysine incorporating genes, the generality of the approach can improve.

The individual and combined rankings are shown in Table 2.

### Hierarchical clustering of PYLIS structures

To assess evolutionary relationships between known pyrrolysine incorporating genes we group the PYLIS regions of these genes into clusters that are similar in structure as follows. We consider all genes that are members of the reciprocal blast clusters which include RefSeq annotated genes. We perform a hierarchical clustering of the genes using a form of neighbor joining (rapidNJ [20]) resulting in a phylogenetic tree that depicts structural conservation relationships. The clustering method relies on a distance measure between a pair of sequences, e.g., derived from a structural alignment. Our clustering does not rely on alignment of predicted structures. Instead, our distance measure is calculated using PMCOMP [19] which is based on alignment of base-pairing probabilities of the sequences. We compute the base-pairing probabilities using the RNAFOLD tool. The distance between two sequences is the inverse of the alignment score, *score*_*A*-*B*_, from PMCOMP:

$$\mathrm{dis}t_{A-B}=\frac{1}{\mathrm{scor}e_{A-B}}.$$

**Figure 3:**
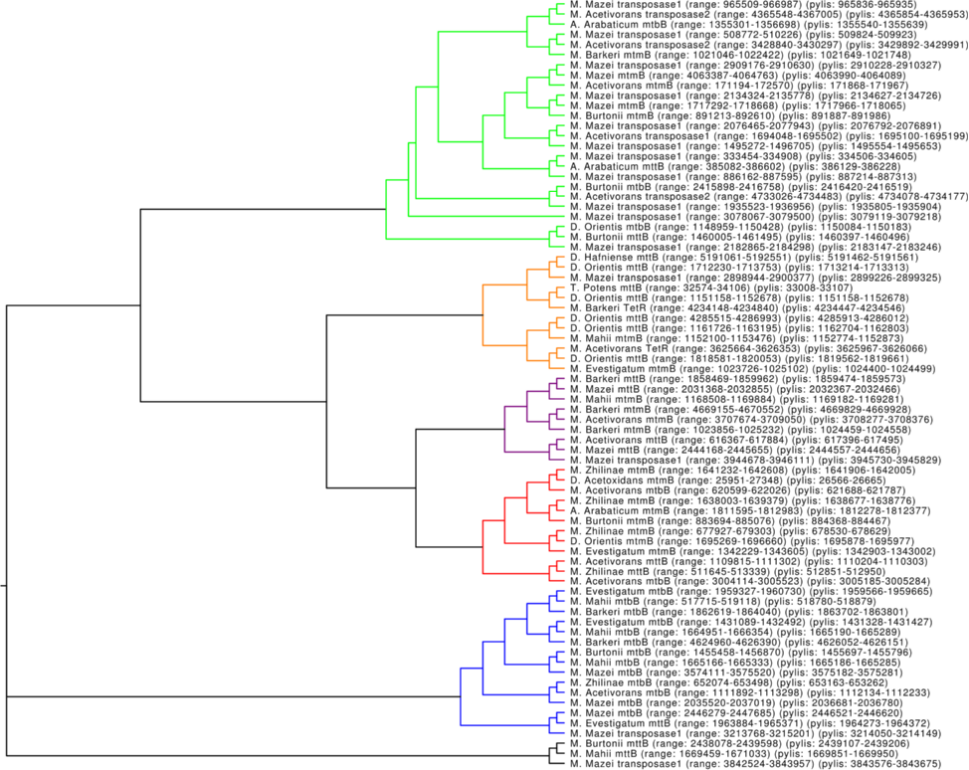
**Structural clustering of PYLIS regions from iORFs in the clusters for known pyrrolysine incorporating genes.** The figure shows that there are certain structural groupings which roughly correspond to gene fami-lies. Almost all transposase genes are in the green cluster (26 sequences), but the cluster also contains sequences from the mtmB (5 sequences), mttB (2 sequences) and mtbB (2 sequences) families. This is also a quite diverse cluster in terms of maximal distance between elements. The orange cluster (12 sequences) predominantly contains mttB genes (8 sequences), but also includes the TetR genes (2 sequences), a single transposase1 gene and a single mtmB gene. The purple cluster (9 sequences) is a mix of mttB genes (4 sequences) and mtmB genes (4 sequences), but includes a single spurious transposase gene. The red cluster (12 sequences) is predominantly mtmB (8 sequences), but includes also two mttB genes and two mtbB genes. The blue cluster (15 sequences) mostly contains mtbB genes (13 sequences) and includes a distant sub-cluster having a mttB gene and a transposase1 gene.

The resulting dendrogram of PYLIS regions is shown in Figure 3.

## Results and discussion

To ensure generalization capability and to minimize model complexity, we systematically assess the ranking features using a criterion of statistical significance. In effect, this assessment leads to a deeper understanding of the factors influencing pyrrolysine translation. We inspect and discuss the list of candidate genes ranked by our method and we discuss structural conservation for known pyrrolysine incorporating genes.

### Ranking of gene clusters

Our method to automatically identify pyrrolysine coding candidate genes is unique in that it utilizes both coding potential, structural conservation, and amino acid conservation. Additionally, we take into account the number of organisms with homologous PYLIS regions as well as the length of up and downstream the potential in-frame UAG.

The ranking is systematically modeled from the known genes taking several factors into account and weighs the different features with respect to the distance of known genes using pyrrolysine (Table 2).

The method has some limitations due to our assumptions. We assume that the PYLIS region is comprised of 100 bases downstream the UAG and that it is well-conserved due to the presumptive presence of a PYLIS structure. Our method cannot detect pyrrolysine containing genes that have divergent PYLIS regions with no significant conservation in homologues and it cannot predict genes with only non-pyrrolysine incorporating homologues.

Our approach is similar to an earlier approach called read-through Similarity Analysis [9]. As in our approach, the authors extract iORFs from candidate genomes and perform a reciprocal blast analysis. The query is a 100 bp window pivoting around the read-through codon. In the case of pyrrolysine incorporating genes this means that the downstream region is shorter than in our case and may not hold the entire PYLIS structure. They calculate an alignment score for the region downstream the read-through codon and a measure of statistical significance by aligning shuffled sequences. Hits with sufficiently high significance are examined further. These hits are expanded using psi-blast and manually checked for non read-through codons lining up with read-though codons. Their driving assumption is that read-through genes will have non read-through homologues.

They identify 34 methyl transferases some of which do not contain UAG in four archaea species (*M. acetivorans, M. burtonii, M. barkeri* and *M. mazei*). We predict only 26 methyl transferases in these genomes. This may be because we enforce more conservative requirements for iORFs and clusters. On the other hand, [9] found only 29 pyrrolysine containing genes among the M. acetivorans and M. mazei while we found 34 among the same two species^f^. A detailed gene by gene comparison is difficult, since their annotations were made on draft genomes and they identify predictions only in terms of their homologues.

Another approach sets out to discover unknown amino acids by conservation of iORFs [10]. The approach is also capable of detecting pyrrolysine incorporating genes. It also begins with iORF extraction and uses blast to detect homologous sequences. The authors use a query (80 bp) centered around the stop codon. Similar to our approach, the blast search results in a number of clusters which is then reduced by pruning rules. Unlike our approach, they only examine clusters with interspecies matches. To distinguish adjacent genes, they exploit the synteny, by looking for blast hits to the N-terminal and the C-terminal of candidates in other genomes. If there are distinct but closely arranged hits in other genomes, this is taken to indicate evidence of adjacent genes rather than a single pyrrolysine incorporating gene. Furthermore, they filter iORFs clusters based on purifying selection, i.e. they prune hits with significantly high incidence of non-synonymous codon usage in the sequences flanking the read-through codon.

As in our approach, they identify clusters for all three methyl transferase families and also a cluster for the TetR family. They do not detect the transposase genes. The methyl transferase clusters they identify have considerably fewer members than what we find. They detect 7 mtmB genes (we detect 19), 7 mtbB genes (we detect 18) and 6 mttB genes (we detect 18). This difference is in part due to the fact that we include more recently published genomes in our search.

However, unlike [9] we do not require non-pyrrolysine homologues in the methyltransferase prediction, and unlike [10] our method can detect genes with only paralogoue conservation within a species. This is reasonable, since some annotated pyrrolysine containing genes are only found in several copies in the same genome.

### Candidate genes

As a result of our method, we obtain a ranking of the clusters in which the known pyrrolysine incorporating genes occur within the top 25 clusters. The ranked list is supplied as Additional file 1. Several other high ranking clusters seem to be false positives, but there are also some interesting candidates.

• An example, which is probably a false positive, is the cluster with rank 11. Although it is conserved in three organisms and is on average 711 bases long, it has the problem that in T. potens it overlaps with a much longer gene in another reading frame. Either the long gene (CRISPR-assosiated protein Cas2) is wrongly annotated, or the short one (CRISPR-associated protein Cas1) “shadows” by incidence the long one. However, in the two other organisms, D. hafniense and D. orientis, the possibility of a direct translation of UAG is more probable since the two annotated genes are in same reading frame and within the predicted iORF.

• An example of a probable gene participating in neutral evolution (drift) is the cluster with rank 7. It consists of only two T. potens genes that are almost identical. However, they are both long and the genes flank another gene (putative anaerobic sulfite) which is in the same reading frame. What has probably happened is that a random mutation has introduced a stop-codon. However, since it is an UAG-codon and the introduction of pyrrolysine does not obstruct the protein, the mutation is conserved in the organism, although it might not introduce an extra advantage. (A similar neutral evolution is believed to occur in known genes of M. acetivorans as in clusters 19 and 23 [21]). This type of pyrrolysine usage does usually not create completely new genes with new functions.

• In cluster 16, a less neutral selection is probable in the evolution towards using UAG in-frame, since it occurs in three different Archaea. The organisms are closely related and the iORF covers a gene (sensory transduction histidine kinase) and a hypothetical protein in the same reading frame in M. acetivorans, a full pseudogene in M. mahii and a shadow part of a sensory transduction histidine kinase in M. barkeri. Likewise, the three methyl transferases are products of selection towards a function of producing methane.

All candidates need to be verified experimentally before they can be determined as pyrrolysine encoding. In addition to this, the clusters containing the three known methyl transferases include several genes annotated as pseudogenes, as two genes, or not at all. Only 21 of the 46 methyl transferases in the 12 investigated organisms are annotated correctly, i.e., as one non-pseudo gene with an in-frame UAG stop codon. These are likely deficiencies in the existing annotations and should be corrected or included after further investigation.

### Structural conservation and the evolution of genes

Our assessment of ranking features indicates that the majority of clusters have a degree of potential structural similarity that is comparable to the clusters for known pyrrolysine genes. Consequently, neither the *f*^*structure*^ feature nor the complex $\sqrt[{3}]{{\hat{f}}^{\mathrm{structure}}\times {\hat{f}}^{\mathrm{diversity}}\times {\hat{f}}^{\mathrm{syn}\_\mathrm{codons}}}$ feature is adequate to recover all the known genes. The known genes seem to have either high structural similarity and low primary sequence diversity (as is the case for the two transposases) or they have low structural similarity but high primary sequence diversity. If we instead consider only the three methyl transferase clusters when calculating feature significance, then the *p*-value for $\mathrm{rank}\left(\sqrt[{3}]{{\hat{f}}^{\mathrm{structure}}\times {\hat{f}}^{\mathrm{diversity}}\times {\hat{f}}^{\mathrm{syn}\_\mathrm{codons}}}\right)$ is 0:012 which is significant within the 0:05 limit^f^. This may suggest that the methyl transferases have important structures although the statistical significance does not necessarily imply biological relevance. It is difficult to say if there is an important structure in the transposases because of the high sequence similarity.

Note that a difference in structures of different clusters does not affect ranking using $\sqrt[{3}]{{\hat{f}}^{\mathrm{structure}}\times {\hat{f}}^{\mathrm{diversity}}\times {\hat{f}}^{\mathrm{syn}\_\mathrm{codons}}}$ or *f*^*structure*^. The *f*^*structure*^ feature does not imply a particular structure for a cluster – it only implies the presence of a high degree of structural similarity within the cluster. Neither of these two features were used to produce the final ranking (see Table 2), which are based only on features that are significant when considering all six known pyrrolysine genes.

The structural clustering of the PYLIS sequences of known pyrrolysine incorporating genes (see Figure 3) reveals relatively compact clusters that roughly correspond to the gene families. It is possible to observe general trends of the clustering, but chance similarities distort the clustering accuracy to an undesired degree and the presence of outliers should not be given too much attention. It is, however, clear that the UAG downstream regions of pyrrolysine incorporating genes do not all share similar structures. There are distinct sub-groupings that may correspond to distinct structures and hence it seems that there are several possible PYLIS structures.

**Figure 4:**
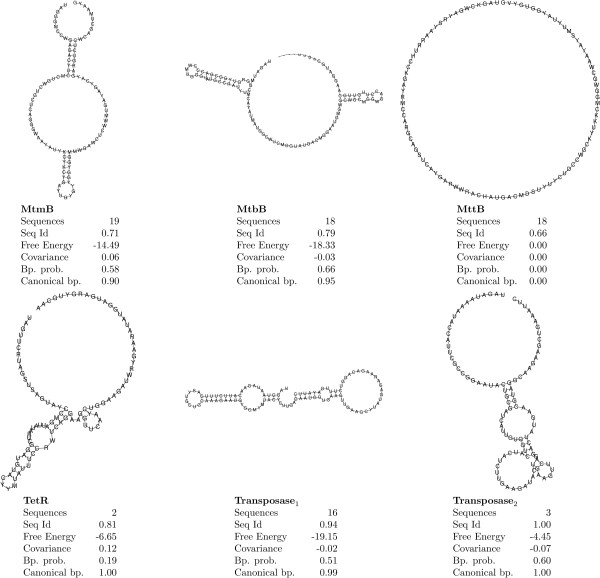
**Predicted consensus structures for each of the known pyrrolysine incorporating clusters.** The consensus sequences are using IUPAC Ambiguity Codes. The consensus structures are predicted through the WAR web service [22] and are based on a variety of methods for structure predictions and alignments integrated through the WAR web service ([23-36]). For each structure, the average pairwise identity, free energy, covariance, base pair probability and percentage canonical base pairs are reported. The consensus structures suggested are not necessarily the correct structures, but reasonable (usually conservative) approximations. The different methods integrated in WAR predict slightly different structures, but the similarities between these are reflected in the consensus structure. It is clear that there are several different structures, but also that mttB does not seem to have any significant structure.

It is possible to create a relatively canonical secondary structure for the PYLIS region of mtmB and mtbB. The mtmB structure has similarities to the previously predicted PYLIS structure [Rfam:RF01982]^g^, but the mtbB structure does not share these similarities. The same region in mttB does not show any sign of common structure. However, since mttB is the gene present in most different organisms and has a high degree of sequence diversity, an elevated variance in the structure is possible. See Figure 4 for predicted consensus structures. The set of predictions from all methods used are included as Additional file 2.

A search using Infernal reveals that using the predicted structures has a positive impact on the recall and precision of detecting the pyrrolysine containing genes mtmB and mtbB, when compared to searching without the predicted structure (data not shown).

Our findings support that pyrrolysine has different ways to evolve in the genomes containing the tRNA^pyl^ as suggested in [21]. For the methyl transferases, a selection for producing methane may have conserved the structures as well as the amino acid residue. In other cases, a neutral evolution is believed to have occured, allowing for a single mutation leading to an in-frame amber stop codon [21]. In our list of candidate genes, a few high ranking candidates had multiple homologues of the UAG in-frame codon either within only one genome or among a few only. These genes are typically conserved within a given species and have even accomplished duplication events. However, as the mutation is not conserved among different species, these may be relatively recent adaptations.

These different gene evolution models constitute a challenge for the approach to select the clusters that represent true positives among clusters of iORFs.

## Conclusions

In this work, we presented a method for predicting pyrrolysine coding genes. The method clusters genes with homologue sequences downstream the in-frame UAG. The clusters are ranked according to observed properties of existing homologous pyrrolysine incorporating genes so that top ranking candidates correspond to known pyrrolysine incorporating gene families or to promising new candidates.

Our method is successful in recovering conserved pyrrolysine containing genes and additionally detects several promising candidates that are not currently annotated. We provide a ranked list of potential pyrroly-sine coding gene candidates.

In addition, our method provides insights into the features that characterise pyrrolysine incorporating genes. We find evidence of conserved structures only within mtmB and mtbB and provide substantiation to suggest that pyrrolysine genes may also arise due to neutral evolution.

## Endnotes

^a^RefSeq annotated genes, except genes where one of the words “pseudo, predicted, putative, unknown, possible, hypothetical or probable” occur in the gene product description.

^b^This is prevalent for instance in M. mazei.

^c^The adaptation corresponds to removing the in-frame UAG codon before decoding.

^d^There are six of clusters that contain known pyrrolysine-incorporating genes.

^e^We calculate cumulative density function using the pnorm command in R.

^f^The species compared were chosen by [9]

^g^P-values are calculated using the same procedure as used in section on feature significance, but with n = 3 and considering only the main rank of the three methyl transferase clusters.

^h^ The RF01982 structure was also only predicted on the basis on mtmB genes. Our consensus structure prediction does not include all the base pairings from RF01982, but mostly agree on the tip of the hairpin. Predictions with MXSCARNA [23] and PETFOLD [37] agree on most base-pairings and have lower free energy scores than the consensus structure.

## Competing interests

The authors declare that they have no competing interests.

## Authors’ contributions

CTH designed and wrote the pipeline, collected the data, performed the data analysis, and participated in the drafting of the manuscript. SZ participated in designing the pipeline, participated in the data analysis, performed the manual analysis of candidate genes clusters and participated in the drafting of the manuscript. HC supervised and financed the study, participated in designing the pipeline, and participated in the drafting of the manuscript. All authors approved the final manuscript.

## Supplementary Material

Additional file 1 **Annotated list of ranked clusters.** The file, supplied as an Excel sheet, includes 25 top-ranked clusters with our manual annotations. Click here for file

Additional file 2 **Complete set of alignments and predicted structures.** This zip file contains a subdirectory for each of the six clusters for known pyrrolysine incorporating genes, where alignments and predicted structures using all methods supported by the WAR web-service are available in fasta format. Click here for file
